# Supplementary material for: Physical health risks of middle-aged people with low social independence: fatal diseases in men, and little attendance to cancer screenings in both sexes
Source: PeerJ. 2023 Feb 20;11:e14904. doi: 10.7717/peerj.14904 (PMC9948749; doi:10.7717/peerj.14904)
Supplement: Supplemental Information 4 [file peerj-11-14904-s004.pdf]

㊙ 政府統計

国民生活基礎調査【世帯票】

（2019年6月6日調査）

厚生労働省 ひと、くらし、みらいのために Ministry of Health, Labour and Welfare

調査員記入欄

地区番号						単位区番号			世帯番号		

この調査は、統計法に基づき国が実施する基幹統計調査です。
調査票情報の秘密の保護に万全を期していますので、ありのままを記入してください。

<記入上の注意>

- **『（世帯票・健康票）記入のしかた』をよくお読みになってから記入してください。**
- **もし記入方法がわからなかった場合は、調査員が受け取りにうかがったときにおたずねください。**
- **選択肢は指示がない場合は、あてはまる番号１つに○をつけてください。**
- **数字は右づめで記入してください。**
- **できるだけ黒のボールペンで記入してください。**

あなたの世帯について、2019年６月６日現在の状況をお答えください。

- **世帯とは、ふだん住居と生計を共にしている人々（世帯員）の集まりをいいます。**
- **世帯員には、旅行や出張などで一時的（３か月以内）に自宅を離れている人や船員など就業場所を移動する人も含みます。**
  また、病院・診療所に入院している人も含みますが、住民登録を病院・診療所に移している人は除きます。さらに、単身赴任や学業で世帯を離れている人、老人福祉施設などの社会福祉施設に入所している人も除きます。

Ⅰ　世帯の状況

質問1　ふだん一緒にお住まいで、生計を共にしている方（世帯員）は、**あなたを含めて何人**ですか。（一時的に不在の方を含みます。）

□□ 人

１人（単独世帯）の場合は、**補問1-1**にもお答えください。

補問1-1　１人（単独世帯）の方の場合は、その状況についてお答えください。

【単独世帯の状況】
1　住み込み、寄宿舎等に居住する単独世帯
2　その他の単独世帯

→ 【単身赴任か否か】
1　単身赴任者である
2　単身赴任者でない

質問2　現在は、単身赴任などで世帯を離れているが、その前は、一緒にお住まいで生計を共にしていた方がいる場合は、**あてはまるすべての番号**に○をつけ、それぞれの人数を記入してください（いない場合は、7に○をつけてください。）。

1　単身赴任で世帯を離れている者がいる → □ 人
2　学業のため世帯を離れている者がいる → □ 人
社会福祉施設に入所している者がいる
3　老人福祉施設に入所している者がいる → □ 人
4　障害者支援施設に入所している者がいる → □ 人
5　3,4以外の社会福祉施設に入所している者がいる → □ 人
6　病院に長期入院している（住民登録を病院に移している。）者がいる → □ 人
7　1～6の者はいない

※　1～6に該当する方は、この調査の世帯員とはなりませんので、**質問1**の人数には含めないでください。

裏面に続きます。

SAMPLE

質問3　お住まいの**住居の種類**と**建て方**についてお答えください。

【住居の種類】
1　持ち家
2　民間賃貸住宅
3　社宅・公務員住宅等の給与住宅
4　都市再生機構・公社等の公営賃貸住宅
5　借間・その他

（1～4 → 建て方へ）

【建て方】
1　一戸建て
2　共同住宅
（マンション、アパート等）

質問4　お住まいの住居の**室数**について、居住用の部屋数（玄関や風呂等は含めないでください。）を記入してください。
また、**床面積**は、玄関や廊下等も含めた住宅全体のおおよその床面積を記入してください。

**【室数】**	**【床面積】**
□□ 室	□□□□．□ ㎡

※　床面積の換算
1坪＝3．3㎡
2畳＝3．3㎡

質問5　**５月中の家計支出総額**（世帯の方全員の支出金額の合計額）を記入してください。

□□□ 万円

※　以下の費用は家計支出には含めないでください。
（税金、社会保険料、事業上の支払い(農家における肥料や農具、商店における商品の仕入れに使った金等)、貯蓄、借金や住宅ローンなどの返済、掛け捨て型以外の生命保険料・損害保険料）

補問5-1　小学校入学前の方がいる場合は、**５月中**の家計支出総額のうち、**育児にかかった費用**を記入してください。

□□ 万 □ 千円

※　育児にかかった費用とは、ミルク代、離乳食代、医療費、保育料、習い事の費用、衣服費、おもちゃ代、その他育児にかかった費用のすべてが入ります。

補問5-2　**５月中**の家計支出総額のうち、別居している親又は子への**仕送りの状況**をお答えください。
仕送りの目的については、**あてはまるすべての番号**に○をつけてください。

【仕送りの状況】
別居している**親**がいる
1　仕送りあり →
2　仕送りなし
3　別居している親はいない

**【仕送りの目的】**	**【仕送り額】**
1　入所・入院費<br>2　その他	□□□ 万円

【仕送りの状況】
別居している**子**がいる
1　仕送りあり →
2　仕送りなし
3　別居している子はいない

**【仕送りの目的】**	**【仕送り額】**
1　学　業<br>2　その他	□□□ 万円

引き続き「Ⅱ　世帯員の状況」についてもお答えください。

SAMPLE

㊙ 政府統計

国民生活基礎調査【世帯票】

（2019年6月6日調査）

厚生労働省 ひと、くらし、みらいのために Ministry of Health, Labour and Welfare

Ⅱ　世帯員の状況

調査員記入欄

地区番号		単位区番号		世帯番号		—	

・すべての世帯員の方について、ひとり一列で記入してください。
・世帯員の記入順序は、**夫婦・親子の関係がある方を順に並べて**記入してください。
・選択肢は指示がない場合は、あてはまる番号１つに○をつけ、数字は右づめで記入してください。

質問	（世帯員番号） 01	（世帯員番号） 02	（世帯員番号） 03
**質問1　最多所得者**<br>調査日前１年間で**所得**（年金、仕送り等を含む。）**が最も多かった方１名**の世帯員番号に○をつけてください。	01	02	03
**質問2　世帯主との続柄**<br>世帯主の配偶者（夫又は妻）の祖父母・兄弟姉妹はそれぞれ「09 祖父母」「10 兄弟姉妹」に含めます。兄弟姉妹の配偶者は「10 兄弟姉妹」に含めます。「配偶者」には、事実上夫婦として生活しているが、婚姻届を提出していない場合も含みます。	⓪① 世帯主<br>02 世帯主の配偶者<br>03 子<br>04 子の配偶者<br>05 孫<br>06 孫の配偶者<br>07 世帯主の父母<br>08 配偶者の父母<br>09 祖父母<br>10 兄弟姉妹<br>11 その他の親族<br>12 その他（親族以外）	02 世帯主の配偶者<br>03 子<br>04 子の配偶者<br>05 孫<br>06 孫の配偶者<br>07 世帯主の父母<br>08 配偶者の父母<br>09 祖父母<br>10 兄弟姉妹<br>11 その他の親族<br>12 その他（親族以外）	02 世帯主の配偶者<br>03 子<br>04 子の配偶者<br>05 孫<br>06 孫の配偶者<br>07 世帯主の父母<br>08 配偶者の父母<br>09 祖父母<br>10 兄弟姉妹<br>11 その他の親族<br>12 その他（親族以外）
**質問3　性**	1 男　2 女	1 男　2 女	1 男　2 女
**質問4　出生年月**	1 明治　4 平成<br>2 大正　5 新元号<br>3 昭和<br>□□ 年 □□ 月	1 明治　4 平成<br>2 大正　5 新元号<br>3 昭和<br>□□ 年 □□ 月	1 明治　4 平成<br>2 大正　5 新元号<br>3 昭和<br>□□ 年 □□ 月
**質問5　配偶者（夫又は妻）の有無**<br>「配偶者」には、事実上夫婦として生活しているが、婚姻届を提出していない場合も含みます。	1 配偶者あり<br>2 未婚<br>3 死別<br>4 離別	1 配偶者あり<br>2 未婚<br>3 死別<br>4 離別	1 配偶者あり<br>2 未婚<br>3 死別<br>4 離別
**質問6　医療保険の加入状況**<br>保険証又は組合員証で確認してお答えください。なお、後期高齢者医療制度に加入している方は、「5」のみに○をつけてください。	国民健康保険 → 1 都道府県・市町村<br>2 組合<br>被用者保険（協会けんぽ、組合健保、共済組合等） → 3 加入者本人<br>4 家族（被扶養者）<br>5 後期高齢者医療制度<br>6 その他	国民健康保険 → 1 都道府県・市町村<br>2 組合<br>被用者保険（協会けんぽ、組合健保、共済組合等） → 3 加入者本人<br>4 家族（被扶養者）<br>5 後期高齢者医療制度<br>6 その他	国民健康保険 → 1 都道府県・市町村<br>2 組合<br>被用者保険（協会けんぽ、組合健保、共済組合等） → 3 加入者本人<br>4 家族（被扶養者）<br>5 後期高齢者医療制度<br>6 その他
**質問7　公的年金・恩給の受給状況**<br>受給している場合、受給している年金等の**すべての番号**に○をつけてください。<br>なお、老齢年金のほか、障害年金、遺族年金なども含めてお答えください。	受給している：<br>01 基礎年金<br>02 基礎年金と厚生年金<br>03 基礎年金と共済年金<br>04 基礎年金と厚生年金と共済年金<br>05 国民年金<br>06 福祉年金<br>07 厚生年金<br>08 共済年金<br>09 恩給<br>10 その他<br>11 受給していない	受給している：<br>01 基礎年金<br>02 基礎年金と厚生年金<br>03 基礎年金と共済年金<br>04 基礎年金と厚生年金と共済年金<br>05 国民年金<br>06 福祉年金<br>07 厚生年金<br>08 共済年金<br>09 恩給<br>10 その他<br>11 受給していない	受給している：<br>01 基礎年金<br>02 基礎年金と厚生年金<br>03 基礎年金と共済年金<br>04 基礎年金と厚生年金と共済年金<br>05 国民年金<br>06 福祉年金<br>07 厚生年金<br>08 共済年金<br>09 恩給<br>10 その他<br>11 受給していない
**小学校入学前の方についてお答えください。**			
**質問8　乳幼児（小学校入学前）の保育状況**<br>日中に保育をしている方及び乳幼児が通所・通園している施設の**すべての番号**に○をつけてください。	1 乳幼児の父母<br>2 乳幼児の祖父母<br>3 認可保育所<br>4 認可外保育施設<br>5 幼稚園<br>6 認定こども園<br>7 その他	1 乳幼児の父母<br>2 乳幼児の祖父母<br>3 認可保育所<br>4 認可外保育施設<br>5 幼稚園<br>6 認定こども園<br>7 その他	1 乳幼児の父母<br>2 乳幼児の祖父母<br>3 認可保育所<br>4 認可外保育施設<br>5 幼稚園<br>6 認定こども園<br>7 その他
**6歳以上の方についてお答えください。**			
**質問9　手助けや見守りの要否**<br>障害や身体機能の低下などで、手助けや見守りを必要としていますか。	手助けや見守りを<br>1 必要としている<br>2 必要としていない → 裏面の質問10へ。15歳未満の方は質問終了です。	手助けや見守りを<br>1 必要としている<br>2 必要としていない → 裏面の質問10へ。15歳未満の方は質問終了です。	手助けや見守りを<br>1 必要としている<br>2 必要としていない → 裏面の質問10へ。15歳未満の方は質問終了です。
**手助けや見守りを必要としている方についてお答えください。**			
**補問9-1　日常生活の自立の状況**<br>最もあてはまる状況の**番号１つ**に○をつけてください。	1 何らかの障害等を有するが、日常生活はほぼ自立しており独力で外出できる<br>2 屋内での生活はおおむね自立しているが、介助なしには外出できない<br>3 屋内での生活は何らかの介助を要し、日中もベッド上での生活が主体であるが座位を保つ<br>4 １日中ベッド上で過ごし、排せつ、食事、着替において介助を要する	1 何らかの障害等を有するが、日常生活はほぼ自立しており独力で外出できる<br>2 屋内での生活はおおむね自立しているが、介助なしには外出できない<br>3 屋内での生活は何らかの介助を要し、日中もベッド上での生活が主体であるが座位を保つ<br>4 １日中ベッド上で過ごし、排せつ、食事、着替において介助を要する	1 何らかの障害等を有するが、日常生活はほぼ自立しており独力で外出できる<br>2 屋内での生活はおおむね自立しているが、介助なしには外出できない<br>3 屋内での生活は何らかの介助を要し、日中もベッド上での生活が主体であるが座位を保つ<br>4 １日中ベッド上で過ごし、排せつ、食事、着替において介助を要する
**補問9-2　期間**<br>補問9-1で答えた自立の状況になってからの期間をお答えください。	1 １月未満<br>2 １～３月未満<br>3 ３～６月未満<br>4 ６月～１年未満<br>5 １～３年未満<br>6 ３～５年未満<br>7 ５～10年未満<br>8 10～20年未満<br>9 20年以上	1 １月未満<br>2 １～３月未満<br>3 ３～６月未満<br>4 ６月～１年未満<br>5 １～３年未満<br>6 ３～５年未満<br>7 ５～10年未満<br>8 10～20年未満<br>9 20年以上	1 １月未満<br>2 １～３月未満<br>3 ３～６月未満<br>4 ６月～１年未満<br>5 １～３年未満<br>6 ３～５年未満<br>7 ５～10年未満<br>8 10～20年未満<br>9 20年以上
**補問9-3　要介護認定の有無**<br>**この質問は40歳以上の方のみお答えください。**<br>実際にサービスを受けているかどうかは問いません。	要介護認定を<br>1 受けている　2 受けていない	要介護認定を<br>1 受けている　2 受けていない	要介護認定を<br>1 受けている　2 受けていない
**主に手助けや見守りをしている方**はどなたかお答えください。（質問9で手助けや見守りを「1 必要としている」と回答した方への質問です。）			
**補問9-4　同別居の状況**<br>主に手助けや見守りをしている方が同居している場合は、**その方の世帯員番号（最上段の番号）を記入してください。**	主に手助けや見守りをしている方は<br>1 同居している　2 同居していない<br>【世帯員番号】→ □□	主に手助けや見守りをしている方は<br>1 同居している　2 同居していない<br>【世帯員番号】→ □□	主に手助けや見守りをしている方は<br>1 同居している　2 同居していない<br>【世帯員番号】→ □□
**補問9-5　主に手助けや見守りをしている方の続柄**<br>主に手助けや見守りをしている方について、**手助けや見守りが必要な方からみた続柄**をお答えください。	1 配偶者<br>2 子<br>3 子の配偶者<br>4 父母<br>5 その他の親族<br>6 事業者（ホームヘルパー等）<br>7 その他（ボランティア・近所の人など）	1 配偶者<br>2 子<br>3 子の配偶者<br>4 父母<br>5 その他の親族<br>6 事業者（ホームヘルパー等）<br>7 その他（ボランティア・近所の人など）	1 配偶者<br>2 子<br>3 子の配偶者<br>4 父母<br>5 その他の親族<br>6 事業者（ホームヘルパー等）<br>7 その他（ボランティア・近所の人など）
**補問9-6　主に手助けや見守りをしている方の性**	1 男　2 女	1 男　2 女	1 男　2 女

裏面に続きます。（15歳未満の方については質問終了です。）

SAMPLE

表面の世帯員番号と同じ列に記入してください。

（世帯員番号）	01	02	03

１５歳以上の方についてお答えください。

質問10　教育

現在、学校に在学しているかどうかお答えください。
「在学中」の方はその学校について、「卒業」の方は最終卒業学校（中途退学をした方はその前の学校）についてお答えください。
・予備校などはここでいう学校には含めません。
・「1 小学・中学」又は「2 高校・旧制中」に〇をつけた方で「1 特別支援学校・特別支援学級」に在学中又は卒業した方はこちらにも〇をつけてください。

1 在学中
2 卒業
3 在学したことがない

1 小学・中学
2 高校・旧制中
3 専門学校
4 短大・高専
5 大学
6 大学院

1 特別支援学校・特別支援学級

質問11　公的年金の加入状況

加入している
20歳以上60歳未満の方は原則として加入しています。

加入していない
20歳未満で仕事をしていない方、すでに老齢年金又は退職年金を受給している方、受給資格があるが受給待ちの方などが該当します。

公的年金に加入している
1 国民年金第１号被保険者（自営業者や学生等）
2 国民年金第２号被保険者（会社員や公務員等）
3 国民年金第３号被保険者（会社員や公務員等の配偶者）
4 公的年金に加入していない

質問12　別居している子の有無

別居している子の有無について、お答えください。

別居している子が
1 いる
2 いない → （質問13へ）

補問12-1　最も近くに住んでいる別居の子の居住場所

1 同一家屋
2 同一敷地
3 近隣地域
4 同一市区町村
5 その他の地域

質問13　５月中の仕事の状況

収入を伴う仕事を少しでもした方は「仕事あり」、まったく仕事をしなかった方は「仕事なし」の中からお答えください。

無給で自家営業の手伝いをした場合や、育児休業や介護休業のため、一時的に仕事を休んでいる場合も「仕事あり」とします。PTA役員やボランティアなど無報酬の活動は「仕事なし」とします。なお、家事には、育児、介護などを含めます。

仕事あり
1 主に仕事をしている
2 主に家事で仕事あり
3 主に通学で仕事あり
4 その他

仕事なし
5 通学
6 家事
7 その他
（質問18へ）

質問13で「1」～「4」（仕事あり）と回答した方についてお答えください。

質問14　１週間の就業日数等

5月20日（月）～26日（日）の1週間に実際に仕事をした日数と時間をお答えください。
なお、複数の仕事をした場合は、すべての合計をお答えください。

【就業日数】
1週間の仕事をした日数 □ 日

【就業時間】
1週間の残業も含めた総時間 □□□ 時間

現在の主な仕事についてお答えください。

質問15　就業開始時期

主な仕事について、その仕事についた時期をお答えください。

1 大正　3 平成
2 昭和　4 新元号
□□ 年 □□ 月

質問16　仕事の内容（職業分類）

主な仕事について、お答えください。

01 管理的職業従事者
02 専門的・技術的職業従事者
03 事務従事者
04 販売従事者
05 サービス職業従事者
06 保安職業従事者
07 農林漁業従事者
08 生産工程従事者
09 輸送・機械運転従事者
10 建設・採掘従事者
11 運搬・清掃・包装等従事者
12 分類不能の職業

質問17　勤めか自営かの別

主な仕事について、お答えください。

01、02、03、04と答えた方は、**補問17-1、17-2**をお答えください。

01 一般常雇者（契約期間の定めのない雇用者）
02 一般常雇者（契約期間が１年以上の雇用者）
03 1月以上1年未満の契約の雇用者
04 日々又は1月未満の契約の雇用者
05 会社・団体等の役員
06 自営業主（雇人あり）
07 自営業主（雇人なし）
08 家族従業者（自家営業の手伝い）
09 内職
10 その他
（質問終了です。）

補問17-1　勤め先での呼称

「労働者派遣事業所の派遣社員」とは労働者派遣法に基づく事業所に雇用され、そこから派遣されている人をいいます。

1 正規の職員・従業員
2 パート
3 アルバイト
4 労働者派遣事業所の派遣社員
5 契約社員
6 嘱託
7 その他

補問17-2　企業規模・官公庁の別

本社・本店や出張所などを含めた企業全体の従業員数をお答えください。
「官公庁」とは、国の機関や地方自治体をいいます。

1 1～4人
2 5～29人
3 30～99人
4 100～299人
5 300～499人
6 500～999人
7 1000～4999人
8 5000人以上
9 官公庁

質問13で「5」～「7」（仕事なし）と回答した方についてお答えください。

質問18　就業希望の有無

就業希望の有無について、お答えください。

収入を伴う仕事を
1 したいと思っている
2 したいと思っていない（質問終了です。）

仕事をしたいと思っている方

補問18-1　どのような形で仕事をしたいと思いますか

最もしたいと思う仕事の形の**番号1つ**に〇をつけてください。
現在仕事を探していない方でも、仕事につくとしたらどのような形で仕事をしたいかをお答えください。

1 正規の職員・従業員
2 パート・アルバイト
3 労働者派遣事業所の派遣社員
4 契約社員・嘱託
5 自営
6 その他

補問18-2　すぐにでも仕事につけますか

すぐに仕事に
1 つける
2 つけない

補問18-3　仕事を探していますか

現在採用結果を待っている方も「探している」に〇をつけてください。

仕事を
1 探している
2 探していない

補問18-4　仕事につけない理由

あてはまるすべての番号に〇をつけてください。

【仕事につけない理由】
1 出産・育児のため
2 介護・看護のため
3 健康に自信がない
4 その他

ご記入ありがとうございました。

SAMPLE
