# Supplementary material for: Physical health risks of middle-aged people with low social independence: fatal diseases in men, and little attendance to cancer screenings in both sexes
Source: PeerJ. 2023 Feb 20;11:e14904. doi: 10.7717/peerj.14904 (PMC9948749; doi:10.7717/peerj.14904)
Supplement: Supplemental Information 5 [file peerj-11-14904-s005.docx]

Comprehensive Survey for Living Conditions

[Household]

(June 6, 2019, survey)

This survey is a fundamental statistical survey conducted by the government in accordance with the Statistics Law.

We take all possible measures to protect the confidentiality of the information in the questionnaire, so please fill in the questionnaire as it is.

<Please fill in the form as it is.

Please read the "How to Fill Out the (Household and Health) Form" carefully before filling out the form.

If you do not know how to fill out the form, please ask the surveyor when you pick it up.

If there are no instructions, please circle the number that applies to you.

Please fill in the numbers right justified.

Please use a black ballpoint pen if possible.

Please indicate the status of your household as of June 6, 2019.

A household is a group of people (household members) who usually live together and make a living.

Household members include those who are away from home temporarily (within 3 months) for travel, business trips, etc., as well as those who move from one place of employment to another (e.g., sailors, etc.).

Also, persons who are hospitalized in hospitals or clinics are included, but those who have their resident registration transferred to the hospital or clinic are excluded. Also excluded are those who are away from their households due to single employment or study, and those who are enrolled in social welfare facilities such as welfare facilities for the aged.

I. Household Situation

Question 1: How many people (household members) do you usually live with and share a livelihood with, including you? (Including those who are temporarily absent.) ）

II. Status of Household Members

・Please fill in the form of all household members in a row.

(Survey conducted on June 6, 2019)

・Please fill in the order of entry of household members in order of those who have a relationship between husband and wife and parents and children.

・ If there are no instructions, circle the number that applies to you, and fill in the numbers right next to each other.

Question 2: Relationship with the head of household

| 01 Head of household  07 Parents of the head of household  02 Spouse of Head of Household  08 Spouse's Parents  03 son  09 Grandparents  04 Spouse of Child  10 Brothers and sisters  05 Sun  11 Other relatives  06 Spouse of grandchildren  12 Others (other than relatives) |
| --- |
| 1 male 2 female |
| 1 Meiji  4 Heisei  2 Taisho  5 New era　Year Month  3 Showa |
| 1 Has a spouse 2 Unmarried  3 Farewell 4 Parting |

Question 9: Need help or watch over

Do you have a disability or a decline in physical function that requires help or supervision ?

Help and watch

1 need 2 does not need

^Question 17-1 Designation at the place of work^

1 Regular employees

2 Parts

3 Part-time jobs

4 Temporary employees at worker dispatching offices

5 Contract employees

6 Commissioning

7 Others

Question 18 Whether or not you wish to work

Please tell us if you would like to work.

1 You want to do a job with an income

2 I don't want to

(End of question.) )

Question 18-2: Can you get a job right away?

Question 18-3 Are you looking for a task?

Question 18-4 Why not do you get the job?

【Reasons why you can't get a job】

1For childbirth and childcare

2 For nursing care and nursing

3 Not confident in health

4 Other
