# Supplementary material for: Physical health risks of middle-aged people with low social independence: fatal diseases in men, and little attendance to cancer screenings in both sexes
Source: PeerJ. 2023 Feb 20;11:e14904. doi: 10.7717/peerj.14904 (PMC9948749; doi:10.7717/peerj.14904)
Supplement: Supplemental Information 6 [file peerj-11-14904-s006.pdf]

秘 政府統計

ひと、くらし、みらいのために 厚生労働省 Ministry of Health, Labour and Welfare

国民生活基礎調査【健康票】

（2019年６月６日調査）

この調査は、統計法に基づき国が実施する基幹統計調査です。
調査票情報の秘密の保護に万全を期していますので、ありのままを記入してください。

〈 **記入上の注意** 〉

・この調査票は、世帯の方全員が**１人１冊**ずつ、記入してください。
・『（世帯票・健康票）記入のしかた』をよくお読みになってから記入してください。
・もし記入方法がわからなかった場合は、調査員が受け取りにうかがったときにおたずねください。
・選択肢はあてはまる番号１つ、又はあてはまるすべての番号に○をつけてください。
・数字は右づめで記入してください。
・ご自分で記入できない方については、ご家族の方、又は介護をしている方が記入を手伝ってください。
・できるだけ黒のボールペンで記入してください。

SAMPLE

質問1　あなたの性・出生年月を記入してください。性・元号は、**あてはまる番号１つ**に○をつけ、出生年月には数字を**右づめ**で記入してください。

性	出　生　年　月
1　男<br>2　女	1　明治　4　平成<br>2　大正　5　新元号<br>3　昭和<br>□□年□□月

調査員記入欄

地区番号		単位区番号		世帯番号	

質問2　あなたは**現在**、病院や診療所に入院中、又は、介護保険施設に入所中ですか。

1　は　い　→　**質問終了です。**
2　いいえ

※　介護保険施設とは、介護医療院、介護療養型医療施設、介護老人保健施設及び介護老人福祉施設をいいます。

質問3　あなたは**ここ数日**、病気やけがなどで体の具合の悪いところ（自覚症状）がありますか。

1　あ　る　　2　な　い　→　**質問4へ**

補問3－1　それは、どのような症状ですか。**あてはまるすべて**の症状名の番号に○をつけてください。その中で最も気になる症状名の番号を番号記入欄に記入してください。

全身症状
- 01　熱がある
- 02　体がだるい
- 03　眠れない
- 04　いらいらしやすい
- 05　もの忘れする
- 06　頭痛
- 07　めまい

眼
- 08　目のかすみ
- 09　物を見づらい

耳
- 10　耳なりがする
- 11　きこえにくい

胸部
- 12　動悸
- 13　息切れ
- 14　前胸部に痛みがある

呼吸器系
- 15　せきやたんが出る
- 16　鼻がつまる・鼻汁が出る
- 17　ゼイゼイする

消化器系
- 18　胃のもたれ・むねやけ
- 19　下痢
- 20　便秘
- 21　食欲不振
- 22　腹痛・胃痛
- 23　痔による痛み・出血など

歯
- 24　歯が痛い
- 25　歯ぐきのはれ・出血
- 26　かみにくい

皮膚
- 27　発疹(じんま疹・できものなど)
- 28　かゆみ(湿疹・水虫など)

筋骨格系
- 29　肩こり
- 30　腰痛
- 31　手足の関節が痛む

手足
- 32　手足の動きが悪い
- 33　手足のしびれ
- 34　手足が冷える
- 35　足のむくみやだるさ

尿路生殖器系
- 36　尿が出にくい・排尿時痛い
- 37　頻尿(尿の出る回数が多い)
- 38　尿失禁(尿がもれる)
- 39　月経不順・月経痛

損傷
- 40　骨折・ねんざ・脱きゅう
- 41　切り傷・やけどなどのけが

- 42　その他

最も気になる症状の番号記入欄　→　[　　]番

補問3－2　最も気になる症状に対して、なんらかの治療をしていますか。**あてはまるすべて**の番号に○をつけてください。

1　病院・診療所に通っている（往診、訪問診療を含む）
2　あんま・はり・きゅう・柔道整復師（施術所）にかかっている
3　売薬をのんだり、つけたりしている
4　それ以外の治療をしている
5　治療をしていない

SAMPLE

質問4　あなたは**現在**、傷病（病気やけが）で病院や診療所（医院、歯科医院）、あんま・はり・きゅう・柔道整復師（施術所）に通っていますか。（往診、訪問診療、補問3−1の症状で通っているものを含む）

1　通っている　　2　通っていない → **質問5へ**

補問4−1　どのような傷病（病気やけが）で通っていますか。**あてはまるすべて**の傷病名の番号に○をつけてください。その中で最も気になる傷病名の番号を番号記入欄に記入してください。

内分泌・代謝障害
- 01 糖尿病
- 02 肥満症
- 03 脂質異常症（高コレステロール血症等）
- 04 甲状腺の病気

精神・神経
- 05 うつ病やその他のこころの病気
- 06 認知症
- 07 パーキンソン病
- 08 その他の神経の病気（神経痛・麻痺等）

- 09 眼の病気
- 10 耳の病気

循環器系
- 11 高血圧症
- 12 脳卒中（脳出血、脳梗塞等）
- 13 狭心症・心筋梗塞
- 14 その他の循環器系の病気

呼吸器系
- 15 急性鼻咽頭炎(かぜ)
- 16 アレルギー性鼻炎
- 17 慢性閉塞性肺疾患（COPD）
- 18 喘息
- 19 その他の呼吸器系の病気

消化器系
- 20 胃・十二指腸の病気
- 21 肝臓・胆のうの病気
- 22 その他の消化器系の病気

- 23 歯の病気

皮膚
- 24 アトピー性皮膚炎
- 25 その他の皮膚の病気

筋骨格系
- 26 痛風
- 27 関節リウマチ
- 28 関節症
- 29 肩こり症
- 30 腰痛症
- 31 骨粗しょう症

尿路生殖器系
- 32 腎臓の病気
- 33 前立腺肥大症
- 34 閉経期又は閉経後障害（更年期障害等）

損傷
- 35 骨折
- 36 骨折以外のけが・やけど

- 37 貧血・血液の病気
- 38 悪性新生物（がん）
- 39 妊娠・産褥（切迫流産、前置胎盤等）
- 40 不妊症
- 41 その他
- 42 不明

最も気になる傷病の番号記入欄 → ［　　］番

６歳未満の方は質問終了です。６歳以上の方は続けてお答えください。

質問5　あなたは**現在**、健康上の問題で日常生活に何か影響がありますか。

1　あ　る　　2　な　い → **質問6へ**

補問5−1　それはどのようなことに影響がありますか。**あてはまるすべて**の番号に○をつけてください。

1　日常生活動作（起床、衣服着脱、食事、入浴など）
2　外出（時間や作業量などが制限される）
3　仕事、家事、学業（時間や作業量などが制限される）
4　運動（スポーツを含む）
5　その他

SAMPLE

質問6　**過去１か月**の間に、健康上の問題で床についたり、普段の活動ができなかった（仕事・学校を休んだ、家事ができなかった等）日数はどれくらいありましたか。日数を**右づめ**で記入してください。

１ ない　２ ある → 合計［　　］日

質問7　あなたの現在の健康状態はいかがですか。**あてはまる番号１つ**に○をつけてください。

１ よい　２ まあよい　３ ふつう　４ あまりよくない　５ よくない

質問8　あなたは現在、サプリメントのような健康食品（健康の維持・増進に役立つといわれる成分を含む、錠剤、カプセル、粉末状、液状などに加工された食品）を食べたり、飲んだりしていますか。

１ はい　２ いいえ

※「サプリメントのような健康食品」に含まれるもの、含まれないものの例については、「記入のしかた」の35～36ページをご参照ください。

12歳未満の方は質問終了です。12歳以上の方は続けてお答えください。

質問9　あなたは**現在**、日常生活で悩みやストレスがありますか。

１ ある　２ ない → **質問10へ**

補問9－1　それは、どのような原因ですか。**あてはまるすべて**の原因の番号に○をつけてください。その中で最も気になる原因の番号を番号記入欄に記入してください。

01	家族との人間関係	12	妊娠・出産
02	家族以外との人間関係	13	育児
03	恋愛・性に関すること	14	家事
04	結婚	15	自分の学業・受験・進学
05	離婚	16	子どもの教育
06	いじめ、セクシュアル・ハラスメント	17	自分の仕事
07	生きがいに関すること	18	家族の仕事
08	自由にできる時間がないこと	19	住まいや生活環境（公害、安全及び交通事情を含む）
09	収入・家計・借金等	20	その他
10	自分の病気や介護	21	わからない
11	家族の病気や介護		

最も気になる悩みやストレスの番号記入欄 →［　　］番

SAMPLE

補問9－2　悩みやストレスを、どのように相談していますか。**あてはまるすべての番号に○をつけてください。**また、最も気になる悩みやストレスについてどのように相談していますか。**あてはまる番号の主なものを２つまで番号記入欄に記入してください。**

01 家族に相談している
02 友人・知人に相談している
03 職場の上司、学校の先生に相談している
04 公的な機関(保健所、福祉事務所、精神保健福祉センター等)の相談窓口(電話等での相談を含む)を利用している
05 民間の相談機関(悩み相談所等)の相談窓口（電話等での相談を含む）を利用している
06 病院・診療所の医師に相談している
07 テレビ、ラジオ、新聞等の相談コーナーを利用している
08 01～07以外で相談している（職場の相談窓口等）
09 相談したいが誰にも相談できないでいる
10 相談したいがどこに相談したらよいかわからない
11 相談する必要はないので誰にも相談していない

最も気になる悩みやストレスの相談状況の番号記入欄→ [　　] [　　] 番

質問10　あなたの**過去１か月**の１日の平均睡眠時間はどのくらいでしたか。**あてはまる番号１つに○をつけてください。**

1 ５時間未満
2 ５時間以上６時間未満
3 ６時間以上７時間未満
4 ７時間以上８時間未満
5 ８時間以上９時間未満
6 ９時間以上

質問11　あなたは**過去１か月**、睡眠によって休養が充分にとれていますか。**あてはまる番号１つに○をつけてください。**

1 充分とれている　2 まあまあとれている　3 あまりとれていない　4 まったくとれていない

質問12　次の(ア)から(カ)の質問について、**過去１か月**の間はどのようであったか、６つの項目それぞれの**あてはまる番号１つに○をつけてください。**

	いつも	たいてい	ときどき	少しだけ	まったくない
(ア) 神経過敏に感じましたか	1	2	3	4	5
(イ) 絶望的だと感じましたか	1	2	3	4	5
(ウ) そわそわ、落ち着かなく感じましたか	1	2	3	4	5
(エ) 気分が沈み込んで、何が起こっても気が晴れないように感じましたか	1	2	3	4	5
(オ) 何をするのも骨折りだと感じましたか	1	2	3	4	5
(カ) 自分は価値のない人間だと感じましたか	1	2	3	4	5

20歳未満の方は質問終了です。20歳以上の方は続けてお答えください。

SAMPLE

質問13　あなたは週に何日くらいお酒(清酒、焼酎、ビール、洋酒など)を飲みますか。
あてはまる番号１つに○をつけてください。

1　毎　　　日	6　ほとんど飲まない
2　週５～６日	7　やめた
3　週３～４日	8　飲まない（飲めない）
4　週１～２日	
5　月１～３日	

（6～8）→ **質問14へ**

（1～5）↓

補問13－1　お酒を飲む日は１日あたり、どのくらいの量を飲みますか。
清酒に換算し、**あてはまる番号１つ**に○をつけてください。

1　１合（180ml）未満	4　３合以上４合（720ml）未満
2　１合以上２合（360ml）未満	5　４合以上５合（900ml）未満
3　２合以上３合（540ml）未満	6　５合（900ml）以上

※ 清酒1合（アルコール度数15度・180ml）は、次の量にほぼ相当
ビール中瓶１本（同５度・500ml）、焼酎0.6合（同25度・約110ml）、ワイン1/4本（同14度・約180ml）、ウイスキーダブル１杯（同43度・60ml）、缶チューハイ1.5缶（同５度・約520ml）

質問14　あなたはたばこを吸いますか。**あてはまる番号１つ**に○をつけてください。

1　毎日吸っている
2　時々吸う日がある
3　以前は吸っていたが１か月以上吸っていない
4　吸わない

（1・2）→ １日に平均して何本くらい吸いますか。

1　10本以下
2　11～20本
3　21～30本
4　31本以上

質問15　あなたは日ごろ、健康のために次のような事柄を実行していますか。
あてはまるすべての番号に○をつけてください。

1　規則正しく朝・昼・夕の食事をとっている
2　バランスのとれた食事をしている
3　うす味のものを食べている
4　食べ過ぎないようにしている
5　適度に運動（スポーツを含む）をするか身体を動かしている
6　睡眠を十分にとっている
7　たばこを吸わない
8　お酒を飲み過ぎないようにしている
9　ストレスをためないようにしている
10　その他
11　特に何もしていない

SAMPLE

質問16　あなたは**過去１年間**に、健診等（健康診断、健康診査及び人間ドック）を受けたことがありますか。

注：次のようなものは健診等には含まれません。
がんのみの検診、妊産婦検診、
歯の健康診査、
病院や診療所で行う診療としての検査

1　ある　　2　ない → **補問16－2へ**

補問16－1　どのような機会に健診等を受けましたか。**あてはまるすべて**の番号に○をつけてください。

※　1～3の各機関が指示する医療機関で受けた場合は、それぞれの機関の番号に○をつけてください。

1　市区町村が実施した健診
2　勤め先又は健康保険組合等（家族の勤め先を含む）が実施した健診
3　学校が実施した健診
4　人間ドック（上記1～3以外の健診で行うもの）
5　その他

【補問16－2は質問16で「2 ない」と答えた方のみお答えください。】

補問16－2　それは、どのような理由で受けなかったのですか。**あてはまるすべて**の番号に○をつけてください。

01　知らなかったから
02　時間がとれなかったから
03　場所が遠いから
04　費用がかかるから
05　検査等(採血、胃カメラ等)に不安があるから
06　その時、医療機関に入通院していたから
07　毎年受ける必要性を感じないから
08　健康状態に自信があり、必要性を感じないから
09　心配な時はいつでも医療機関を受診できるから
10　結果が不安なため、受けたくないから
11　めんどうだから
12　その他

次頁へ続きます。

SAMPLE

※　質問17、質問18、質問19のがん検診については、健診等（健康診断、健康診査及び人間ドック）の中で受診したものも含みます。

質問17　あなたは**過去１年間**に、下記の３つのがん検診を受けましたか。それぞれの検診についてお答えください。また、受診した検診ごとに、どのような機会に受診したのかお答えください。

胃がん検診(バリウムによるレントゲン撮影や内視鏡（胃カメラ、ファイバースコープ）による撮影など)<br>１　受けなかった　　２　受けた →	どのような機会に検診を受けましたか。<br>**あてはまるすべて**の番号に○をつけてください。<br>１　市区町村が実施した検診<br>２　勤め先又は健康保険組合等（家族の勤め先を含む）が実施した検診<br>３　その他
肺がん検診(胸のレントゲン撮影や喀痰(かくたん)検査など)<br>１　受けなかった　　２　受けた →	どのような機会に検診を受けましたか。<br>**あてはまるすべて**の番号に○をつけてください。<br>１　市区町村が実施した検診<br>２　勤め先又は健康保険組合等（家族の勤め先を含む）が実施した検診<br>３　その他
大腸がん検診(便潜血反応検査(検便)など)<br>１　受けなかった　　２　受けた →	どのような機会に検診を受けましたか。<br>**あてはまるすべて**の番号に○をつけてください。<br>１　市区町村が実施した検診<br>２　勤め先又は健康保険組合等（家族の勤め先を含む）が実施した検診<br>３　その他

質問18　あなたは**過去２年間**に、胃がん検診を受けましたか。また、どのような機会に受診したのかお答えください。

胃がん検診(バリウムによるレントゲン撮影や内視鏡（胃カメラ、ファイバースコープ）による撮影など)<br>１　受けなかった　　２　受けた →	どのような機会に検診を受けましたか。<br>**あてはまるすべて**の番号に○をつけてください。<br>１　市区町村が実施した検診<br>２　勤め先又は健康保険組合等（家族の勤め先を含む）が実施した検診<br>３　その他

20歳以上の女性の方は続けてお答えください。

質問19　あなたは**過去２年間**に、下記の２つのがん検診を受けましたか。それぞれの検診についてお答えください。また、受診した検診ごとに、どのような機会に受診したのかお答えください。

子宮がん（子宮頸がん）検診(子宮の細胞診検査など)<br>１　受けなかった　　２　受けた →	どのような機会に検診を受けましたか。<br>**あてはまるすべて**の番号に○をつけてください。<br>１　市区町村が実施した検診<br>２　勤め先又は健康保険組合等（家族の勤め先を含む）が実施した検診<br>３　その他
乳がん検診(マンモグラフィ撮影や乳房超音波(エコー)検査など)<br>１　受けなかった　　２　受けた →	どのような機会に検診を受けましたか。<br>**あてはまるすべて**の番号に○をつけてください。<br>１　市区町村が実施した検診<br>２　勤め先又は健康保険組合等（家族の勤め先を含む）が実施した検診<br>３　その他

ご記入ありがとうございました。

SAMPLE
