# Supplementary material for: Physical health risks of middle-aged people with low social independence: fatal diseases in men, and little attendance to cancer screenings in both sexes
Source: PeerJ. 2023 Feb 20;11:e14904. doi: 10.7717/peerj.14904 (PMC9948749; doi:10.7717/peerj.14904)
Supplement: Supplemental Information 7 [file peerj-11-14904-s007.docx]

Comprehensive Survey for Living Conditions [Health]

(Survey as of June 6, 2019)

This survey is a fundamental statistical survey conducted by the government in accordance with the Statistics Law. We take all possible measures to protect the confidentiality of questionnaire information, so please fill in the questionnaire as it is. Please fill in the questionnaire as it is.

| < Notes on filling out the form >  Please fill out one copy of this questionnaire for each person in your household.  Please read the "How to Fill Out (Household and Health Form)" carefully before filling out the form.  If you do not know how to fill out the form, please ask the surveyor when you pick it up.  If you do not understand how to fill out the form, please ask the surveyor when you pick it up.  Please circle one number that applies to you or all the numbers that apply to you.  If you are unable to fill out the form by yourself, please have a family member or caregiver help you fill it out.  Please use a black ballpoint pen if possible. |
| --- |

Question 1: Please fill in your gender and date of birth. For gender/era, please add a circle to one of the applicable numbers and write the number in the right column for the year of birth.

| sex | Date of birth |
| --- | --- |
| 1 male  2 female | 1 Meiji  4 Heisei  2 Taisho  5 Era Year Month New  3 Showa |

Investigator Entry Fields

|  | | | | | | | | | | | |
| --- | --- | --- | --- | --- | --- | --- | --- | --- | --- | --- | --- |
| District number |  |  |  |  |  | Unit Ward Number |  |  | Household number |  |  |

- 1 -

Question 2: Are you currently hospitalized in a hospital or clinic, or in a long-term care insurance facility?

| 1 Yes  2 No |
| --- |

Question 4: Are you currently attending a hospital, clinic (doctor's office, dentist's office), or anma, acupuncture, moxibustion, or judo therapist (treatment center) for an injury or illness (disease or injury)? (Including house calls, home visits, and those attended for the symptoms of supplemental Q3-1)

| 1 Going  2 Not going through |
| --- |

Question ~~4-1~~: What kind of illness or injury are you suffering from? Please circle the number of all the injuries or illnesses that apply to you. Please write the number of the injury or illness that concerns you the most in the space provided.

| 01 Diabetes  15 Acute nasopharyngitis (cold)  32 Diseases of the kidneys  02 Obesity  16. Allergic rhinitis  33 BPH  03 Dyslipidemia  17. Chronic obstructive pulmonary disease  34 Menopause or postmenopausal disorders  04 Thyroid Diseases  18 Gasp  35 Fractures  05 Depression  19 Other respiratory systems  36 Injuries other than fractures  06 Dementia  20 Diseases of the stomach and duodenum  37 Anemia and blood diseases  07 Parkinson's disease  21 Diseases of the liver and gallbladder  38 Malignant neoplasms  08 Other Neurological Diseases  22 Other digestive systems  39 Pregnancy and puerperal diseases  09 Eye Diseases  23 Dental diseases  40 Infertility  10 Ear diseases  24. Atopic dermatitis  41 Others  11. Hypertension  25 Other skin diseases  42 Unknown  12 Stroke (cerebral hemorrhage, etc.)  26 Gout  13 Angina pectoris and myocardial infarction  27. Rheumatoid arthritis  14 Other circulatory systems  28. Arthropathy  29. Stiff neck disease  30 Low back pain  31 Osteoporosis  Fill in the number of the symptom that concerns you the most. |
| --- |

- 6 -

Question 16: Have you had any medical examinations (physical examinations, medical checkups, or physical examinations) in the past year? Have you had any medical checkups in the past year?

| 1 Yes  2 No |
| --- |

Question 16-1: On what occasions did you receive health checkups? Please add a circle to all applicable numbers.

* If you receive the service at a medical institution instructed by each institution of 1 ~ 3, please add a circle to the number of each institution.

| 1 Health checkups conducted by municipalities  2. Health checkups conducted by employers or health insurance societies, etc. (including family members' workplaces)  3 Health checkups conducted by the school  4 Comprehensive medical checkup (medical examinations other than those 1 ~ 3 above)  5 Others |
| --- |

Please answer supplemental question 16-2 only if you answered "2 no" to question 16].

16-2

What was the reason why you did not take the course? Please circle all that apply.

| 01 Because I didn't know  08 Because I was confident in my health and didn't feel the need  02 Because I couldn't get an hour    03 Because the place is far away  09 Because you can visit a medical institution whenever you are worried  04 Because it costs  05 Because I am worried about examinations (blood collection, stomach camera, etc.)  10 I don't want to receive it because I'm worried about the outcome    06 Because I was going to a medical institution at that time  11 Because I'm sorry  12 Others    07 Because I don't feel the need to take it every year |
| --- |

- 7 -

- 7 -

Continue to the next page.

Question 17: Have you had any of the following three cancer screenings in the past year? Please answer each question. For each screening, please indicate the occasion for which you were examined.

| Gastric cancer screening (X-ray with barium or endoscope (gastric camera, fiberscope), etc.)  1 Didn't receive  2 Received | What opportunities did you get screened?  Please add a circle to all applicable numbers.  1 Medical examination conducted by municipalities  2. Medical examinations conducted by employers or health insurance societies, etc. (including family members' workplaces)  3 Others |
| --- | --- |
| Lung cancer screening (chest X-ray, sputum examination, etc.)  1 Didn't receive  2 Received | What opportunities did you get screened?  Please add a circle to all applicable numbers.  1 Medical examination conducted by municipalities  2. Medical examinations conducted by employers or health insurance societies, etc. (including family members' workplaces)  3 Others |
| Colon cancer screening (fecal occult blood reaction test (stool examination))  1 Didn't receive  2 Received | _３_  What opportunities did you get screened?  Please add a circle to all applicable numbers.  1 Medical examination conducted by municipalities  2. Medical examinations conducted by employers or health insurance societies, etc. (including family members' place of employment)  3 Others |

Question 18: Have you had stomach cancer screenings in the past two years?

Also, please tell us what kind of occasion you received a medical examination.

| Gastric cancer screening (X-ray with barium or endoscope (gastric camera, fiberscope), etc.)  1 Didn't receive  2 Received | What opportunities did you get screened?  Please add a circle to all applicable numbers.  1 Medical examination conducted by municipalities  2. Medical examinations conducted by employers or health insurance societies, etc. (including family members' workplaces)  3 Others |
| --- | --- |

If you are a woman over the age of 20, please continue to answer.

Question 19: Have you had the following two cancer screenings in the past two years? Please answer about each medical examination. Also, please answer what kind of occasion you received for each medical examination.

| Uterine cancer (cervical cancer) screening (uterine cytology, examination, etc.)  1 Didn't receive  2 Received | What opportunities did you get screened?  Please add a circle to all applicable numbers.  1 Medical examination conducted by municipalities  2. Medical examinations conducted by employers or health insurance societies, etc. (including family members' place of employment)  3 Others |
| --- | --- |
| Breast cancer screening (mammography, breast ultrasound (echo) examination, etc.)  1 Didn't receive  2 Received | What opportunities did you get screened?  Please add a circle to all applicable numbers.  1 Medical examination conducted by municipalities  2. Medical examinations conducted by employers or health insurance societies, etc. (including family members' workplaces)  3 Others |

Thank you for taking the time to fill out this form.

- 8 -

- 8 -
